# The Role of *TuACO* Gene Family in Response to Biotic and Abiotic Stresses in *Triticum urartu*

**DOI:** 10.3390/genes16111259

**Published:** 2025-10-25

**Authors:** Min Li, Xiaoting Liu, Shuo Wang, Xinhai Wang, Pu Gao, Takele Weldu Gebrewahid, Peipei Zhang, Zaifeng Li

**Affiliations:** 1 School of Agriculture and Animal Husbandry Engineering, Zhoukou Polytechnic, Zhoukou 466000, China; 2 College of Plant Protection, Hebei Agricultural University, Baoding 071001, China; 17732468772@163.com (X.L.);; 3 College of Agriculture, Aksum University-Shire Campus, Shire 314, Ethiopia

**Keywords:** *Triticum urartu*, *ACO* gene family, abiotic stress, biotic stress, gene structure, expression pattern

## Abstract

Background: Ethylene is one of the most important plant hormones. *ACC oxidase* (*ACO*) plays a vital role in ethylene synthesis and responses to biotic and abiotic stresses in plants. However, its function in *Triticum urartu* remains unclear. This study aims to systematically identify the members of the *TuACO* gene family to elucidate its response characteristics and functions under biotic and abiotic stresses. Methods: Through homologous alignment, phylogenetic evolution analysis, and investigations of gene structure and promoter cis-elements, a total of eight *TuACO* genes were identified in the *T. urartu* genome based on their homology to *OsACO* and *AtACO* protein sequences. Results: These genes were classified into five *ACO* subfamilies and distributed across chromosomes 1A, 4A, 5A, 6A, and 7A. *TuACO* gene families contained 0–3 introns and 1–4 exons. The protein sequence contains 10 different conservative motifs. QRT-PCR expression analysis revealed that the transcript levels of *TuACO5a*, *TuACO5b*, and *TuACO3a* were significantly upregulated at 6 and 24 h after infection with powdery mildew, a biotic stress. Under boron deficiency, an abiotic stress, the expression of *TuACO6* and *TuACO1b* increased, whereas the expression of *TuACO5b* and *TuACO3b* was markedly induced under high-boron conditions. Conclusions: These results demonstrate that *TuACO* genes exhibit functional diversification in response to biotic and abiotic stresses, which lays the foundation for elucidating their gene functions.

## 1. Introduction

Ethylene, one of the most important gaseous hormones, plays an important role in biotic and abiotic stresses responses in plants. The content of ethylene is lower under normal conditions but dramatically elevated during abiotic or biotic stress responses, where downstream genes regulate through the corresponding ethylene signaling pathway, thereby stimulating and initiating a series of cellular responses [1]. For instance, it has been reported that ethylene levels rise rapidly in plants under drought stress [2].

*ACC oxidase* (*ACO*) and methionine are key enzymes in the ethylene synthesis and precursors of the ethylene biosynthesis pathways, respectively [3]. Initially, S-adenosyl-L-methionine (SAM) synthase catalyzes the conversion of methionine into SAM under the action of ATP [4]; then, *1-aminocyclopropane-1-carboxylic acid* (*ACC*) does so in the presence of *ACC* synthase (*1-aminocyclopropane-1-carboxylate* (*ACC*) *synthase* (*ACS*)) [5,6,7]. Finally, *ACC oxidase* (*1-aminocyclopropane-1-carboxylic acid oxidase*, *ACO*) catalyzes the conversion of *ACC* into ethylene [8,9]. The cloning of the *ACO* gene has been widely reported in dicotyledonous plants [10,11,12,13], while progress has been slow in monocotyledonous plants. For instance, seven *ACO* genes were identified in the barley genome using the *ACO* gene protein sequence of rice [14]. Additionally, *SIACO1*, as the first identified *ACO* gene, has led to the subsequent identification of 9, 13, 5, and 7 *ACO* genes in the rice, maize, Arabidopsis, and apple genomes, respectively, through its protein sequence. Phylogenetic analysis indicates that these genes are diverged from a common ancestral gene [15].

There are limited reports on the identification and functional studies of *ACO* genes in wheat and its progenitor species, although *ACO* genes play an important role in ethylene synthesis and responses to biotic and abiotic stresses. Few examples of the reports include the following: *TaACO1*, from the wheat cultivar Shanrong 3, had a negative regulatory role in the response to salt stress in *Arabidopsis thaliana* [16]; and *TuACO3* from the *T. urartu* wheat, in interaction with TuMYB46L, regulated ethylene synthesis and participated in powdery mildew defense response [17]. Therefore, using *T*. *urartu* as the experimental material to identify more *ACO* genes and analyze their functions can deepen our understanding of its stress tolerance mechanisms. In this study, based on the protein sequences of *ACO* genes from rice and *Arabidopsis thaliana*, *ACO* genes in *T. urartu* wheat will be identified and analyzed for their gene structure, phylogeny, and response patterns to biotic and abiotic stresses. This research will lay the foundation for further investigation into the functions of *TuACO* genes.

## 2. Materials and Methods

### 2.1. Gene Identification and Evolutionary Analysis

According to Hoben’s established research [15], the rice genome contains nine identified *ACO* genes (*OsACO1*-*OsACO7*), with *OsACO3* further classified into three subtypes: *OsACO3a*, *OsACO3b*, and *OsACO3c*. In contrast, the Arabidopsis genome comprises five *ACO* genes (*AtACO1*-*AtACO5*). For this study, *ACO* protein sequences were obtained from the Rice Genome Database (http://rice.uga.edu) and the Arabidopsis Genome Database (https://www.arabidopsis.org/), while the *T. urartu ACO* gene sequence was sourced from the Wheatomics platform (http://wheatomics.sdau.edu.cn). Using the *ACO* gene family profile (PF51471) from the Pfam database (http://pfam.xfam.org/) as the seed sequence with an E-value cutoff of <1 × 10^−5^, candidate *ACO* proteins were predicted from *T. urartu* sequences using HMMER 3.0 (http://www.hmmer.org/) under default parameters. Following the removal of incomplete sequences, conserved domain validation was performed using the SMART online tool (http://smart.embl.heidelberg). The molecular weights and isoelectric points of *TuACO* proteins were analyzed via the ProtParam online platform (https://web.expasy.org), while the subcellular localization of *TuACO* family members was predicted using Wolfpsort (https://www.genscript.com). Phylogenetic analysis was conducted with MEGA7 software, applying 1000 bootstrap replicates while maintaining default settings for all other parameters.

### 2.2. Gene Structure, Promoter, Protein Conserved Motif Analysis, Chromosomal Localization, and Expression Patterns

Gene structures were analyzed using GSDS (http://gsds.cbi.pku.edu.cn), while protein conserved motifs were identified with MEME (http://meme-suite.org/tools/meme), configured to detect 10 motifs with lengths of 10–100 amino acids. Cis-acting elements in promoters were predicted using PlantCARE (http://bioinformatics.psb.ugent.Be/webtools/plantcare/html/) and visualized with TBtools (v1.098). Chromosomal locations were mapped using MapInspect. Following Zhang et al. [17], we obtained *TuACO* gene expression data under powdery mildew induction from Wheatomics (http://wheatomics.sdau.edu.cn) to analyze expression patterns.

### 2.3. Material Handling

*T. urartu* wheat was collected from the Department of Plant Pathology, Hebei Agricultural University, China. The inoculation of powdery mildew was conducted referring to the method described by Zhang et al. [17]. The wheat seeds were germinated hydroponically at 22 °C under 16 h of light or 8 h of darkness, followed by inoculation during the one-leaf growth stage. First, the leaves were lightly misted with water. Then, they were uniformly and thoroughly rubbed with seedlings infected with powdery mildew pathotype E09. Samples were collected for qRT-PCR analysis at 0, 6, and 24 h after inoculation with the powdery mildew pathogen.

High- and low-boron stress treatments were conducted using the method described by Leaungthitikanchana et al. [18]. Five days after germination, *T. urartu* wheat seedlings were transplanted in 2 L brown plastic bottles to be incubated with Hoagland’s culture solution containing 20 nM and 1 m boric acid for 7 days (pH:5.6–6.0), respectively, with artificial climatic chamber conditions of 16 h/day of light, 20–25 °C, and 60–70% relative humidity. The experimental work was set up with a control and three replications for each treatment.

### 2.4. QRT-PCR Analysis

Total RNA was extracted from *T. urartu* wheat seedlings and reverse-transcribed into cDNA. SYBR green was used as a fluorescent quantitative dye for quantitative analysis in an ABI ViiA7 (Applied Biosystems, Foster City, CA, USA) instrument. The primers were synthesized by Beijing Qingke Biotechnology Co., Ltd. (Beijing, China). (Table 1). The amplification program was pre-denaturation at 95 °C for 6 min, denaturation at 95 °C for 10 s, and renaturation at 60 °C for 1 min, for 40 cycles. The quantitative fluorescence results were analyzed by 2^−∆∆Ct^ using β-Actin as an internal reference.

## 3. Results

### 3.1. Identification and Physicochemical Properties Analysis of TuACO Gene Family in T. urartu

Based on the protein sequences of nine *OsACO* genes in rice and five *AtACO* genes in *Arabidopsis thaliana*, a total of eight *TuACO* genes were identified by comparing the *T. urartu* wheat protein database in Wheatomics (http://wheatomics.sdau.edu.cn) using the Blast P tool (Table 2), including two of *TuACO1*, *TuACO3*, and *TuACO5* each, and one of *TuACO4* and *TuACO6* each. Compared with *ACO* genes in rice and Arabidopsis, *TuACO2* and *TuACO7* were not identified in the *T. urartu* genomic sequences of the eight *TuACO* genes, which varied in length from 927 to 1535 bp, with coding sequence length ranging from 927 to 996 bp, and coding amino acid number ranging from 293 to 331. The isoelectric points of the eight *TuACO* genes were 4.84–6.56, and the molecular weights of the proteins were 33.55–36.47 kD. All the eight *TuACO* proteins were hydrophilic proteins located in the cytoplasm.

### 3.2. Clustering Analysis of TuACO Genes

**Figure 1:**
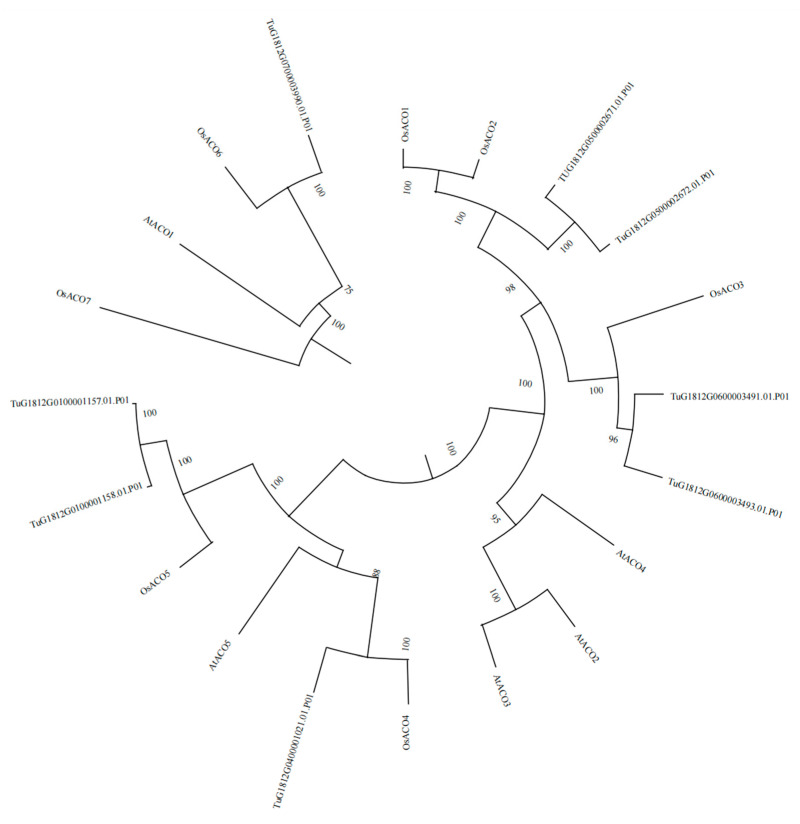
Analysis of *ACO1* gene evolution.

Relationship analysis of the eight *TuACO* genes in *T. urartu*, nine *OsACO* genes of rice, and five *AtACO* genes of *Arabidopsis thaliana* was performed. Their protein sequences were used to construct an evolutionary tree (Figure 1). The clustering results showed that *TUG1812G0500002671.01* and *TUG1812G0500002672.01* clustered with *OsACO1* and *OsACO2*; *TuG1812G0600003491.01* and *TuG1812G0600003493.01* clustered with *OsACO3*; *TuG1812G0400001021.01* clustered with *OsACO4*; *TuG1812G0100001157.01* and *TuG1812G0100001158.01* clustered with *OsACO5*; *TuG1812G0700003990.01* clustered with *OsACO6*; *AtACO2*, *AtACO3*, and *AtACO4* formed a distinct cluster; while *OsACO7* constituted a separate cluster. Therefore, *TUG1812G0500002671.01*, *TuG1812G0500002672.01*, *TuG1812G0600003491.01*, *TuG1812G0600003493.01*, *TuG1812G0700003990.01*, *TuG1812G0400001021.01*, *TuG1812G0100001158.01*, and *TuG1812G0100001157.01* were designated as *TuACO1a*, *TuACO1b*, *TuACO3a*, *TuACO3b*, *TuACO6*, *TuACO4*, *TuACO5a*, and *TuACO5b*, respectively. These results indicate that *T*. *urartu* exhibits a closer phylogenetic relationship with rice, while showing a more distant relationship with *Arabidopsis thaliana*. Additionally, an ortholog of *OsACO7* appears to be absent in the *T*. *urartu* genome.

### 3.3. Chromosomal Localization of TuACO

**Figure 2:**
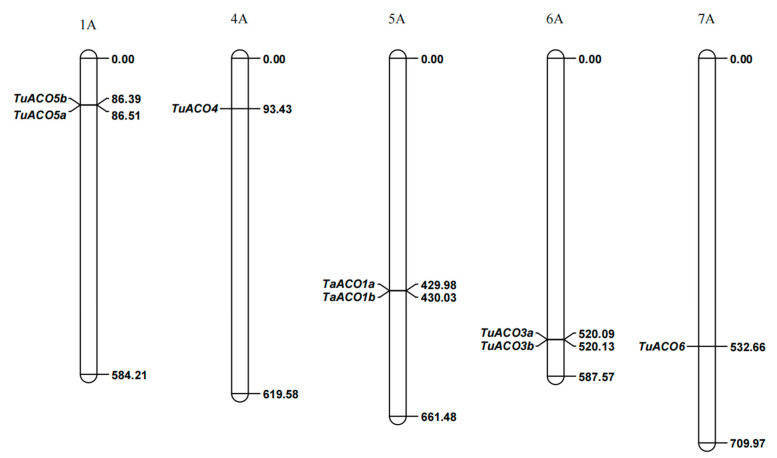
Chromosome location of *TuACO* genes.

Based on the physical positions of *TuACO* genes, eight *TuACO* genes were mapped to five chromosomes (Figure 2). Specifically, *TuACO5a* and *TuACO5b* were located on chromosome 1A; *TuACO4* was located on chromosome 4A; *TuACO1a* and *TuACO1b* were located on chromosome 5A; *TuACO3a* and *TuACO3b* were located on chromosome 6A; and *TuACO6* was located on chromosome 7A. The results showed that the distribution of *TuACO* genes across the chromosomes appeared to be non-uniform. The Ka/Ks ratio (non-synonymous substitution rate/synonymous substitution rate) can reflect a gene’s evolutionary pattern and the selective pressure it has experienced during evolution. In this study, Ka/Ks ratios were used to assess the selection pressure acting on *TuACO5a*/*TuACO5b*, *TuACO1a*/*TuACO1b*, and *TuACO3a*/*TuACO3b* during their evolutionary history. The Ka/Ks values of *TuACO5a*/*TuACO5b*, *TuACO1a*/*TuACO1b*, and *TuACO3a*/*TuACO3b* were 0.10, 0.13, and 0.36, respectively. These results indicated that *TuACO5*, *TuACO2*, and *TuACO1* underwent strong purifying selection throughout their evolutionary process.

### 3.4. The Structure and Protein Conserved Motifs of TuACO

**Figure 3:**
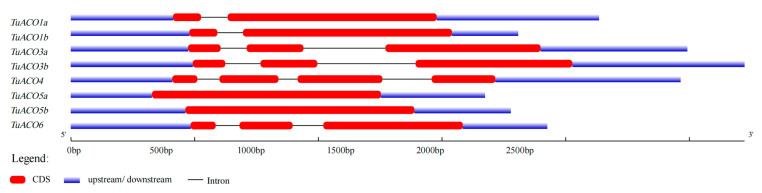
Intron and exon organization of *TuACO*.

**Figure 4:**
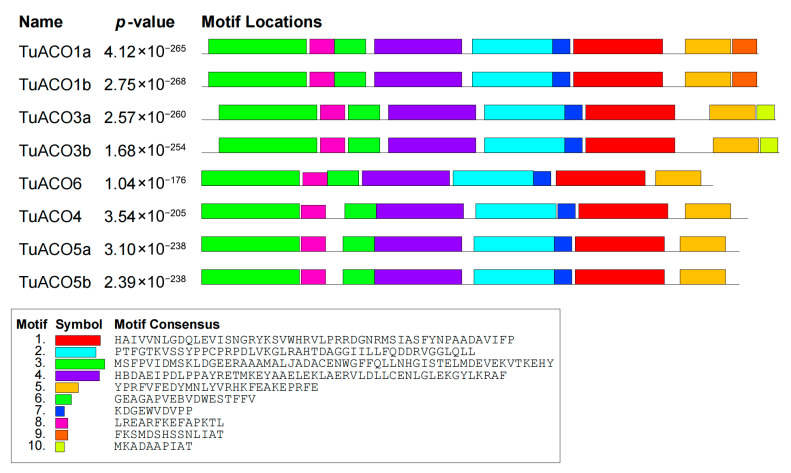
Conserved motif analysis of *TuACO* protein.

The *TuACO* gene structure analysis revealed that the number of introns and exons varied significantly. The number of introns ranged from 0 to 3, while the number of exons ranged from 1 to 4 (Figure 3). *TuACO4* contained three introns and four exons; *TuACO3b*, and *TuACO6* each contained two introns and three exons; *TuACO1a* and *TuACO1b* each contained two exons and one intron; and *TuACO3a*, *TuACO5a* and *TuACO5b* each contained one exon with no intron. These findings indicate substantial structural variation among *TuACO* genes. On the other hand, the protein conserved motif analysis revealed that the *TuACO* protein family contained a total of 10 different conserved motifs, with all eight genes containing motifs 1–8, whereas motif 9 was present in *TuACO1a* and *TuACO1b*, and motif 10 was only present in *TuACO3a* and *TuACO3b* (Figure 4). The functional annotation also showed that motifs 1, 5 and 7 belonged to the 2OG-Fe (II) oxidase superfamily and were typical domains of the *ACO* gene family. Motif and domain analyses reveal that *TuACO* gene sequences are relatively conserved.

### 3.5. Promoter Cis-Acting Primitive Analysis

**Figure 5:**
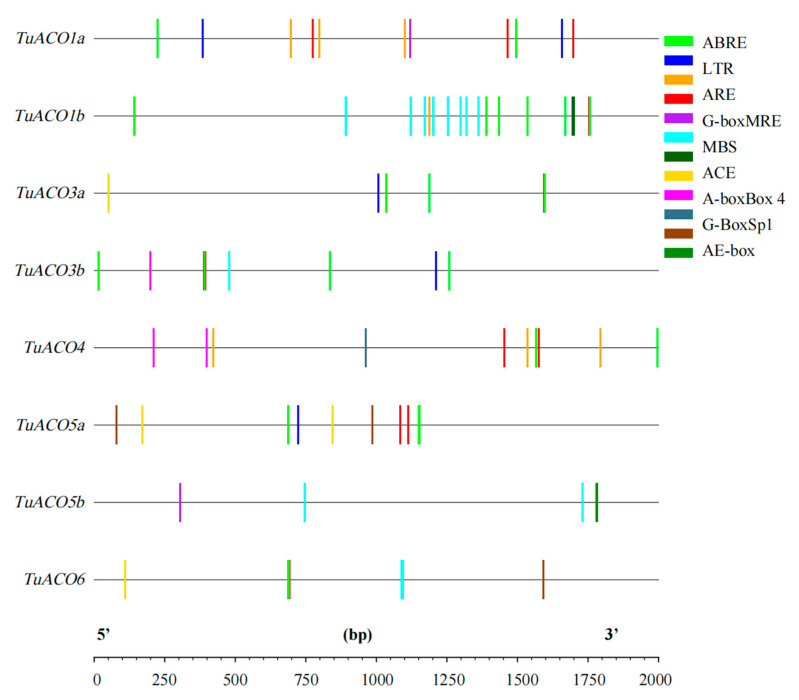
Cis-elements analysis in the promoter of *TuACO* gene.

**Figure 6:**
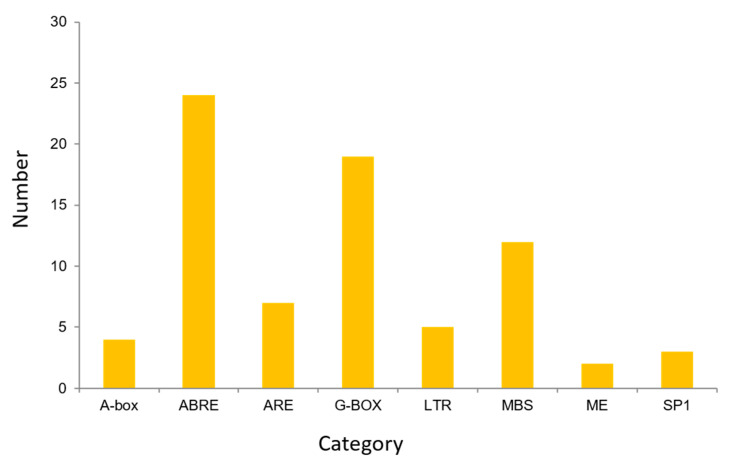
Cis-elements categories in *TuACO* gene promoter.

The cis-element analysis was conducted on the 2000 bp sequence upstream of the start codon of the *TuACO* gene using the online analysis software PlantCARE (Figure 5). The results indicated that the predicted promoters include ABRE (ABA response), LTR (low-temperature stress), ARE (anaerobic induced correlation), MBS (myb transcription factor binding site), G-box, ACE, MRE, Sp1, and Box4 (photoresponsive element). This result demonstrates the gene’s association with a stress response. ABA-responsive elements are the most abundant, followed by light-responsive elements such as G-box (Figure 6). Among the 8 *TuACO* genes, *TuACO1b* contained 16 cis-acting elements, with the ABA response element and myb transcription factor binding sites being the most abundant, while the *TuACO6* and *TuACO5b* had the fewest, with only 4 cis-acting elements (Figure 5). The results suggest potential functional divergence among different *TuACO* genes.

### 3.6. TuACO Response to Biotic Stress

**Figure 7:**
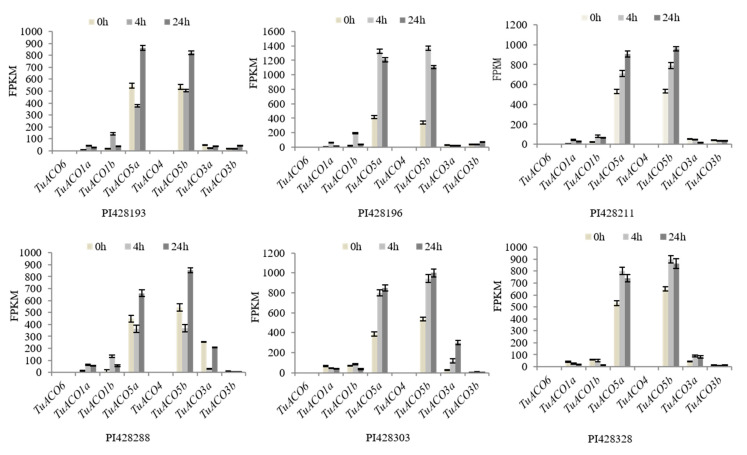
*TuACO* response to powdery mildew.

In order to analyze the response of *TuACO* gene to biological stress, transcriptome expression data of the *TuACO* genes in six *T. urartu* wheat varieties infected by powdery mildew pathogen was obtained from the Wheatomcis website (http://wheatomics.sdau.edu.cn) [17]. The results showed that the expression patterns of the eight *TuACO* genes were generally similar across the six varieties. At 0 h post-inoculation (hpi), 4 hpi, and 24 hpi with powdery mildew, *TuACO5a* and *TuACO5b* consistently exhibited the highest expression levels, while *TuACO6*, *TuACO3b*, and *TuACO4* showed the lowest expression levels, respectively. The expression levels of different genes varied among different varieties. For example, in variety PI428196, the expression levels of *TuACO5a* and *TuACO5b* were significantly upregulated by 4 hpi. In contrast, in varieties PI428193 and PI42888, their expression levels decreased by 4 hpi. In three varieties PI428193, PI428196, and PI428288, *TuACO1b* was significantly upregulated by 4 hpi, while in the other three varieties, the changes were relatively minor. Specifically, *TuACO5a* was downregulated in PI428288 4 hpi (Figure 7). The results indicate that *TuACO5a* and *TuACO5b* are involved in the powdery mildew stress response in *T. urartu*.

**Figure 8:**
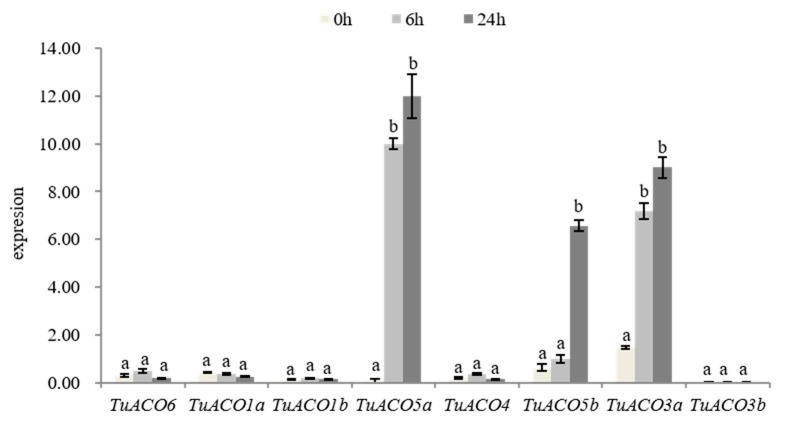
Expression of *TuACO* after infection by powdery mildew. Different letters represent statistical difference *p* < 0.05.

At 0, 6, and 24 hpi, the expression levels of 8 *TuACO* genes were analyzed using quantitative real-time PCR (qRT-PCR). The qRT-PCR analysis validated the transcriptomic data, showing consistent expression trends for all eight *TuACO* genes under the conditions of β-actin normalization and primer efficiencies >95% [17]. Following the inoculation of powdery mildew at 6 and 24 h, the expression levels of *TuACO5a*, *TuACO5b*, and *TuACO3a* were significantly upregulated, whereas no significant differences were detected in the other 5 genes (Figure 8). These findings revealed that *TuACO5a*, *TuACO5b*, and *TuACO3a* were involved in the response of *T. urartu* to powdery mildew. This finding is consistent with the transcriptomic analysis results.

### 3.7. TuACO Response to Abiotic Stress

**Figure 9:**
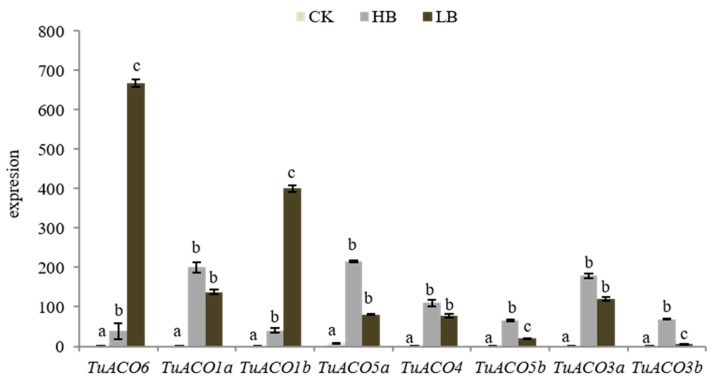
Expression of *TuACO* under high B and low B. Different letters represent statistical difference *p* < 0.05.

The findings demonstrate that *ACO1* plays a critical role in plant stress adaptation [15]. Boron is an essential micronutrient in plant growth and development. Elevated boron levels induce toxicity that impairs normal development, while boron deficiency causes infertility in plants. Analysis of the expression of *TuACO* in *T. urartu* under high- and low-boron stresses revealed the consistent upregulation of all eight genes under both boron-excess and boron-deficient conditions (Figure 9). Under boron-deficient conditions, *TuACO6* and *TuACO1b* exhibited markedly higher expression levels than under boron-excess conditions, indicating a highly significant differential expression. In contrast, *TuACO5b* and *TuACO3b* exhibited the opposite pattern, showing markedly higher expression under high-boron conditions compared to low-boron conditions, and these differences were also highly significant. For *TuACO1a*, *TuACO5a*, *TuACO4*, and *TuACO3a*, there was no significant difference in the expression levels between the high- and low-boron conditions. The results demonstrated functional divergence among *TuACO* genes under boron stress: *TuACO6* and *TuACO1b* respond to boron deficiency, while *TuACO5b* and *TuACO3b* are induced under high-boron conditions.

## 4. Discussion

### 4.1. The TuACO Genes Exhibit Significant Structural Diversity During Evolution While Maintaining Conserved Sequence Architecture

The *ACO* genes belong to the heme-dependent dioxygenase (2OGD) superfamily. Genomic analyses revealed the presence of 9, 13, 5, 7, and 7 *ACO* genes in rice (*Oryza sativa*), maize (*Zea mays*), Arabidopsis (*Arabidopsis thaliana*), apple (*Malus domestica*), and barley (*Hordeum vulgare*), respectively. Notably, three isoforms of *OsACO3* exist in rice [14,15]. Based on the *ACO* protein sequences from rice and Arabidopsis, this study identified eight *TuACO* genes in the *T. urartu* genome. Gene structure analysis revealed that *TuACO* genes contain 0–2 introns and 1–4 exons, indicating structural divergence during evolution. Clustering analysis demonstrated that *TuACO1*, *TuACO3*, and *TuACO5* each have two subtypes, consistent with the *OsACO3* pattern in rice, suggesting gene duplication events in *ACO* evolution. Phylogenetic analysis classified *T. urartu TuACO* genes into five subgroups, compared to six subgroups for rice *OsACO* and three for Arabidopsis *AtACO* genes, revealing higher variation diversity in *T. urartu* and rice than in Arabidopsis, which aligns with previous findings [18]. Protein motif prediction confirmed that all eight *TuACO* genes contain multiple characteristic sequences belonging to the 2OG-Fe(II) oxygenase superfamily. Notably, *TuACO1a*/*TuACO1b* and *TuACO3a*/*TuACO3b* possess motifs 9 and 10, respectively, unlike *TuACO4*, *TuACO6*, *TuACO1a* and *TuACO1b*, demonstrating the conservation of core motifs and structural domains during evolution.

### 4.2. The Distribution of Core Promoter Elements Varies Significantly Among Different TuACO Genes

In rice, *OsACO1*, *OsACO2*, *OsACO3*, *OsACO5*, and *OsACO7* are induced by light, ethylene, auxin, cytokinin, gibberellin, and abscisic acid to participate in diverse biological developmental processes and abiotic stress responses [19,20,21]. Promoter *cis*-acting element analysis revealed that *TuACO* gene promoters contained multiple stress-associated core regulatory elements, including the hormone-responsive element, ABRE; low-temperature stress element, LTR; and light-responsive elements, G-box, ACE, MRE, Sp1, and Box 4 (Figure 5). MYB transcription factors, known for their broad involvement in plant development and stress regulation, were found to have abundant binding sites in *TuACO* promoters. Additionally, anaerobic response elements (ARE) were identified in this trial, and the presence of multiple elements in the promoter is consistent with the *TuACO* gene’s broad involvement in responses to both biotic and abiotic stresses. Among the eight *TuACO* genes, *TuACO1b* contains up to 16 cis-acting elements, with the highest abundance of ABA-responsive elements and MYB transcription factor binding sites. In contrast, *TuACO6* and *TuACO5b* possess only four elements each. This differential distribution of core promoter elements among *TuACO* genes aligns with their involvement in diverse stress responses.

### 4.3. Functional Divergence of TuACO Genes in Response to Biotic and Abiotic Stresses

Ethylene, as a gaseous plant hormone, plays a vital role in the regulation of plant abiotic stress adaptation [22,23]. The *ACO* is the key gene to the conversion of *ACC* to ethylene [24]. In deepwater rice, the levels of *OsACO1* transcripts and enzyme activity were increased by flooding [25]. In etiolated seedlings, the expression levels of *OsACO3* and *OsACO2* were affected by auxin and ethylene [20]. Increasing the expression levels of *LeACO1* and *LeACO4* effectively enhances the brassinosteroid stress response in peach (*Prunus persica*) [18]. *TaACO1* negatively regulates the response of salt stress in *Arabidopsis thaliana* [13]. *TuACO3* plays a significant role in the regulation of powdery mildew resistance [17]. The expression of *OsACO2* in rice increases upon infection with Magnaporthe oryzae, enhancing ethylene biosynthesis and consequently improving resistance to rice blast [26]. Following powdery mildew (*Blumeria graminis*) inoculation, *TuACO3* expression increases across diverse *T. urartu* accessions, correlating with elevated ethylene levels. CRISPR-mediated *TuACO3* knockout reduces both ethylene content and powdery mildew resistance. In this study, the expression levels of *TuACO5a*, *TuACO5b*, and *TuACO1b* were upregulated at 0 h post-powdery mildew inoculation (hpi) and at 4 h and 24 h post-inoculation. For instance, *TuACO6*, *TuACO3b*, and *TuACO4* do not appear to be involved in the response to the powdery mildew pathogen. Further validation of expression patterns demonstrated consistent transcriptional profiles at 0, 6, and 24 h post-inoculation with powdery mildew, aligning with previously reported findings [17].

Boron is an essential nutrient element for plant growth and development [18,27]. This study analyzed the expression profiles of 8 *TuACO* genes under high-boron and low-boron stress. The results showed that all 8 *TuACO* genes were upregulated under both high-boron and low-boron conditions, but they exhibited different responses to high-boron and low-boron stress. Under low-boron conditions, *TuACO6* and *TuACO1b* displayed higher expression levels, indicating their sensitivity to low-boron stress. Under high-boron conditions, *TuACO5b* and *TuACO3b* exhibited higher expression levels, indicating their sensitivity to high-boron stress. The expression levels of *TuACO1a*, *TuACO5a*, *TuACO4*, and *TuACO3a* increased under both high-boron and low-boron conditions, though the differences were not statistically significant, suggesting that these four genes are involved in the response to both high-boron and low-boron stress. The differential expression patterns of various *TuACO* genes under high-boron and low-boron stress imply that changes in their coding sequences may lead to functional divergence; however, this hypothesis requires further experimental validation.

In this study, eight *TuACO* genes were identified in *T. urartu*, and their functions in response to biotic and abiotic stresses were validated. Given that *T. urartu* serves as the A-genome donor of common wheat, this research will establish an important theoretical foundation and provide valuable genetic resources for future disease resistance and stress tolerance breeding.

## Figures and Tables

**Table 1:** Primer sequences for qRT-PCR.

Gene Name	Upstream Primer	Downstream Primer	Amplicon Length
*TuACO1a*	GCTTCGTCGTTCCCGATCAT	GCCCTTTGGTCATCTTCTCCA	166
*TuACO1b*	AACCTCGGCGACCAGC	CGGGTTGTAGAAGGATGCGA	113
*TuACO3a*	CCACCACTACAGGCAAGTGA	CAAAGGCCCGCTTCAGGT	122
*TuACO3b*	GTCGGAGATCGAGAAGCTGG	GAGGCCGTCAACCAGGTC	169
*TuACO6*	GAGACACTTGCGCTTCTCCA	TTGAGGTGACTTTGTCGGGA	190
*TuACO4*	TGAAGAAGCTGGCGGAGAAG	GCCGTAGAAAGGCTCGAAGT	113
*TuACO5a*	GGACTACGTGTTCGGGGATT	GCCTCGAATCTTGGCTCCTT	56
*TuACO5b*	CAAGGTCAGCCACTACCCAC	GCACGTCCAGCCACTCG	149

**Table 2:** Identification and physicochemical properties of *TuACO* gene in *Triticum urartu*.

Gene	ID	Physical Position	Genome (bp)	CDS (bp)	Protein (aa)	Intron	Exon	Weight	Isoelectric Point	Hydrophobicity	Subcellular Localization
*TuACO1a*	TUG1812G0500002671.01	429986928–429989061	1067	960	319	1	2	35.69	5.27	−0.545	cytoplasmic translation
*TuACO1b*	TuG1812G0500002672.01	430039786–430041136	1063	960	319	1	2	35.69	5.29	−0.167	cytoplasmic translation
*TuACO3a*	TuG1812G0600003491.01	520096987–520099477	1426	990	329	2	3	36.47	5.17	−0.111	cytoplasmic translation
*TuACO3b*	TuG1812G0600003493.01	520135882–520138604	1535	996	331	2	3	36.47	5.16	−0.333	cytoplasmic translation
*TuACO6*	TuG1812G0700003990.01	532663043–532664967	1102	882	293	2	3	33.55	6.56	−0.945	cytoplasmic translation
*TuACO4*	TuG1812G0400001021.01	93438404–93440867	1307	942	313	2	4	34.87	4.84	−0.645	cytoplasmic translation
*TuACO5a*	TuG1812G0100001158.01	86510694–86512367	927	927	308	0	1	34.48	4.97	−0.833	cytoplasmic translation
*TuACO5b*	TuG1812G0100001157.01	86396262–86398038	927	927	308	0	1	34.40	4.87	−0.833	cytoplasmic translation

## Data Availability

The original contributions presented in the study are included in the article, further inquiries can be directed to the corresponding author.
